# Testing and refining an approach to identifying health and social care needs in probation

**DOI:** 10.1177/02645505241232128

**Published:** 2024-03-10

**Authors:** Coral Sirdifield, Thomas Parkhouse, Charlie Brooker, Graham Law

**Affiliations:** University of Lincoln, UK; University of London, UK; University of Lincoln, UK

**Keywords:** Probation, health, healthcare, social care, needs assessment

## Abstract

We piloted an approach to identifying the health and social care needs of people on probation using a survey consisting of validated screening tools and key additional questions. We share findings from our analysis of the sample data, showing that there is a high complexity of needs in this population, with 65.4% of participants reporting at least one unmet need. We also explore the acceptability of this approach to identifying needs being used in routine probation practice and make recommendations about how identification and recording of needs could be approached and further researched in the future.

## Background

11Addressing the health and social care needs of people on probation would be beneficial both to the individuals concerned and to the wider society through reductions in health inequalities and re-offending (Revolving Doors Agency, 2017; Richards, 2020). Probation staff perform a role in linking people on probation to health services. For example, playing a critical role ‘in signposting and supporting access to appropriate local mental health treatment’ (HMPPS and NPS, 2019: 11).

For staff to perform this role well sufficient and appropriate services need to be available to meet people’s needs, together with clear pathways to access them. This was recognised in the National Probation Service (NPS) Health and Social Care Strategy, which, when the research was conducted, offered the most up-to-date description of probation’s ambitions in relation to improving the health of people on probation.^1^ For example, the strategy detailed an ambition to ‘influence commissioning processes, where possible, by providing accurate data to demonstrate the prevalence of need’ (HMPPS and NPS, 2019: 8) and to ‘engage with local service providers to improve pathways into mental health treatment and/or services’ (HMPPS and NPS, 2019: 11). Similar ambitions have since been stated in the latest partnership agreement for improving the quality of services for people in prison and those subject to statutory supervision by the probation service in the community. Here, the government state an aim to ‘improve the quality of data and intelligence collection(s) and enable better data-sharing between partners to support the needs analysis of people in our care and support the development of effective health outcome measures’ (HM Government and NHS England, 2023: 18).

1Recording health and social care needs data and the extent to which any identified needs are met by existing service provision as a routine part of probation practice could enable the development of national datasets that could be used to evidence the current needs of the probation population and long-term health trends. However, currently, there is a lack of systematically routinely collected data on the health and social care needs of this population. Moreover, we know that people on probation often struggle to access the care that they need, with for example, those with a combination of substance misuse and mental health problems being bounced between services (HM Government and NHS England, 2023; Richards, 2020: 25; Sirdifield et al., 2019b). Whilst probation practitioners do record some data on the health and social care needs of people on probation on systems like OASys, this is not always done consistently or in a format that is helpful for sharing with health and social care partners. Guidance on conducting needs assessments of this population shows that such recording is often based on yes/no self-report data that are not validated by established screening tools (Richards, 2020). Additionally, some validated screening tools may be completed at the pre-sentence report (PSR) stage (e.g., by liaison and diversion teams). Ideally, the current fields within probation systems would be replaced with measures that are more meaningful to health and social care partners as well as being relevant to probation practice. Furthermore, sufficient resources should be allocated to the probation service to enable such data to be systematically recorded without overloading practitioners. In addition, improvements should be made to ensure that data recorded at the PSR stage are easily reportable and able to be shared at the aggregate level to inform commissioning decisions.

In this study, we aimed to improve the measurement, understanding and recording of health and social care needs of people on probation by piloting a research-informed approach to identifying needs and recording whether they have been met. This took the form of a short survey consisting of validated screening tools and key additional questions. Ultimately, we hoped that this would provide an approach that could be employed nationally and could be used to create reports on the needs of people on probation that could be shared with local Health and Wellbeing Boards to consider within Joint Strategic Needs Assessments to inform commissioning decisions, highlighting where needs are being met, and where there may be gaps in service provision.

In addition, we sought to investigate the relationship between certain demographics and levels of need. Public Health England guidance states that ‘as in the general population, older people can have more severe and complex health needs, that are often combined with social care needs’ (Richards, 2020: 18). Research by Cadet (2021) suggests that this effect can be exacerbated by years spent in prison and experiences prior to prison resulting in people ageing ‘ten years faster than their counterparts in the community’ (Cadet, 2021: 122).

Research investigating the relationship between needs and ethnicity is limited and comparison of findings between studies is problematic due to differences in methodological approaches. Two studies from the U.S. report findings in this area. Graves et al.’s (2023) study of adults in community supervision showed a greater risk of substance use for white people, whilst Lurigio et al., 2003 found that white people on probation were less likely to report past-month drinking and past-year illicit drug use than black people on probation. However, it is difficult to draw conclusions about the current U.K. probation population from these sources.

Research into rurality and health often suggests that health is better in rural areas, with people in urban areas likely to be at higher risk of some health problems, e.g., some psychiatric disorders (Allan et al., 2017; Weich et al., 2006). However, findings are mixed and the rural population is ageing. Whilst health may generally be better in rural areas, access to healthcare is generally worse (Local Government Association and Public Health England, 2017).

We wish to answer the following research questions:

1. What health and social care needs do people on probation have?
2. What do people on probation’s patterns of service access look like?
3. To what extent are the health and social care needs of people on probation met by existing service provision?
4. Does the prevalence of needs and/or the extent to which they are met vary by age, ethnicity or rurality?
5. What proportion of people on probation completed the survey independently and what proportion completed it with the assistance of staff within probation? What, if any impact did this have on responses?
6. How acceptable is this approach to identifying needs to probation staff and what might facilitate it becoming a routine part of probation practice?

## Methods

Ethics permission for the study was gained from the University of Lincoln (2021_6947) and the National Research Committee (2021-124). Key ethical issues included firstly, ensuring that all participants provided informed consent. To enable this, all participants received an information sheet written in plain English that detailed what participation involved, and the associated risks, burdens and benefits as well as information on data storage and the right to withdraw. Staff could read this to potential participants as needed and a video version was also available. These resources made it clear that participation was voluntary, and there would be no negative impact from declining to take part. In terms of risks and burdens, we kept the survey short and recognised that thinking about unmet health needs could be distressing. Each participant was provided with details of support services. It was also important that participants were clear that whilst names would not be recorded when data from the survey were entered for analysis, at the data collection stage probation staff would look at their answers. If a risk to self or others was indicated by their answers this would be discussed with the participant, and other services may be contacted for support.

### Survey construction

After consulting the literature and a Health Leads Group within the probation service, we designed a short survey of health and social care needs, the extent to which they are met, and patterns of service access. The survey was piloted by the Revolving Doors Agency with five people with lived experience of the criminal justice system who timed how long completing the survey took and provided feedback in a focus group. Response times ranged from 7–10 min, suggesting an acceptable survey length. Changes were made to improve the phrasing and to provide a wider range of response options in some sections of the survey.

The final survey consisted of questions on demographics (gender, ethnicity and age) to enable comparison of needs by these variables; past diagnoses; service use and health and social care needs. The latter were investigated using four validated screening tools which were selected on the basis that they were brief, suitable for incorporation into probation systems for completion by people in the criminal justice system and probation practitioners, known to have good levels of validity and reliability, would indicate severity of need in a meaningful way for probation’s health and social care partner agencies, and would produce scores that could inform decisions around onward referral or signposting to services.

Firstly, the Alcohol Use Disorders Identification Test (AUDIT-C) – a short-form tool consisting of three questions designed to provide an initial screen for alcohol misuse issues and to identify those who might benefit from onward referral for support (Bush et al., 1998). The measure generates a total score between 0 and 12. The first question is ‘how often do you have a drink containing alcohol?’ Any participant answering ‘never’ to this question, even those leaving the remaining two items blank, score 0 overall (Bush et al., 1998; Liskola, et al., 2018). There is variation within the literature in terms of the recommended cut-off score for this tool, from three or more in the original validation paper (Bush et al., 1998), to 4+ for men and 3+ for women in a later study (Bradley et al., 2007), and 6+ to identify hazardous drinking in a prison population (Pape et al., 2021). The National Institute for Health and Care Excellence and the U.K. Government Office for Health Improvement and Disparities both recommend a score of 5+ as a positive screen indicating consumption at a level that puts health at risk,^2^ and a score of 11-12 as indicating possible dependence. It is suggested that people scoring this should contact their GP. We used these latter scores in our analysis as we believed this would be meaningful for probation’s healthcare partners.

Secondly, the Drug Abuse Screening Test (DAST-10) was used to screen for drug misuse. This is a 10-item screening tool designed to identify those who may require further interventions with total scores ranging from 0 to 10 (Bohn et al., 1991). A score of 3 or above suggests at least a moderate level issue with drug misuse and was used as the cut-off score in the analysis. As with the AUDIT-C, the initial question asks, ‘have you used drugs other than those required for medical reasons?’ As such, any participant answering no to this question scores 0 overall. The total score can be grouped into five levels of severity: 0 indicates no problems; 1-2 indicates low level; 3–5 indicates moderate level; 6–8 indicates substantial level; whilst 9-10 indicates severe level.

Thirdly, the shortened 10-item version of the Clinical Outcomes in Routine Evaluation (CORE-10; Barkham et al., 2013) was used to screen for current psychological distress. The CORE-10 is used in U.K. primary care mental health settings to screen for current distress and to establish the need for onward referral. Total scores can range from 0 to 40. A cut-off score of 6+ can be used to indicate any current psychological need that would benefit from referral to primary care, and a score of 10+ as indicating a current psychological need that would benefit from referral to secondary care (O’Connor and Morris, 2019). Scores can also be grouped according to level of severity: 0–5 indicates a healthy range; 6–9 indicates a low level; 10–14 indicates a mild level; 15–19 indicates a moderate level; 20–24 indicates moderate to severe; whilst 25+ indicates severe levels of psychological distress.^3^

Finally, the survey included the Camberwell Assessment of Need Short Appraisal Schedule (CANSAS-P; Trauer et al., 2008). This lists 22 potential needs covering domains such as mental health, physical health, and social care. The participants are asked to state whether each item is a met need (an area that is not a serious problem due to the support that they are receiving), an unmet need (an area that remains a problem despite any support received), or not a need. Participants can also choose an ‘I don’t want to answer’ option. Participants in the survey had scores calculated for total number of met needs, total number of unmet needs, and total number of needs overall (met and unmet combined).

All percentages were rounded up to one decimal place and are reported as valid percentages unless indicated otherwise. The participants were also asked to state whether they had completed the survey alone or with the help of probation staff. If completed with help, this was further broken down to determine whether the help was required due to COVID-19 restrictions, or because the participant had difficulty reading the survey.

### Participants

Two probation regions were selected on the basis that they had the capacity to support the study, could provide adequate sample sizes, and would include a Probation Delivery Unit (PDU) with an above-average proportion of ethnic minorities when compared to the national caseload to maximise chances of gathering data from these groups. We selected two urban and two rural PDUs (as defined by the Office for National Statistics urban rural classification maps overlaid onto the PDUs) to collect data from.

Within each PDU, probation staff including frontline practitioners and members of engaging people on probation teams were asked to recruit any adult on probation in the community who they believed had sufficient understanding of English and capacity to provide informed consent. This was done over a three-month period in 2022, which was extended to four months in one region. The survey could be completed in three ways: (a) self-completion at a face-to-face meeting with the member of staff reading the responses straight after the survey was completed, (b) staff-assisted completion at a face-to-face meeting, and (c) staff-assisted completion over the telephone or an online platform (if an appropriate private space was available to the participant).

At the start of the data collection period, there were a total of 7853 people on probation in the community in the participating PDUs. Practitioners were instructed to remind potential participants about completing the survey twice over the following month if needed unless they had previously indicated that they did not wish to take part.

### Missing data

When creating the summary scores for the four screening tools included in the survey, we did not calculate summary scores for participants who failed to complete all items on the AUDIT-C (*n* = 2), DAST-10 (*n* = 2), and CANSAS-P (*n* = 7). Here, the summary scores were recorded as missing values. For the CORE-10, it is recommended that when just one item is missing, the mean score of the nine other items for that participant should be calculated and then multiplied by 10 to generate the summary score.^4^ Therefore, we adhered to this procedure for the two participants with one item missing. We did not calculate summary scores for participants with more than one missing item on the CORE-10 (*n* = 3).

### Comparison between groups

To investigate the relationship between needs and age, participants were grouped as either younger (below 50 years old) or older (50 years and above). To investigate the relationship between ethnicity and levels of need, participants were grouped as white or ethnic minority. To determine whether rurality was related to the level of need observed among our sample, participants were grouped as reporting to probation offices in either urban or rural areas.

### Acceptability

We aimed to conduct semi-structured interviews with 5–10 staff that had administered the survey to explore how acceptable they thought the approach was and what might facilitate it becoming a routine part of probation practice. Interviews were transcribed verbatim and analysed by two researchers using thematic analysis (Braun and Clarke, 2006).

## Results

### Demographics

The overall response rate was poor (*n* = 67). Three surveys (all from one PDU) were less than 50% complete. These were removed before analysis. In addition, as just three participants stated that they were female and two preferred not to say, we have chosen to focus our analysis on the remaining 59 male participants.

The 59 participating males had a mean age of 36.9 years (SD = 12.44). Almost 80% of participants reported their ethnicity as white, whilst 8.5% selected Asian/Asian British, 8.5% selected black/African/Caribbean/black British, and 3.4% selected mixed/multiple ethnic backgrounds. Nearly 80% were aged below 50 years, and 17% were aged 50 years and above. Two participants did not report their age (Table 1). Thus, those aged 50+ and those reporting their ethnicity as white were slightly over-represented in our sample when compared to the overall probation caseload.

Regarding rurality, 64.4% reported to probation offices in urban areas, whilst 35.6% reported to offices in rural areas. 7.4% were Approved Premises residents at the time of data collection.

### What health and social care needs do people on probation have?

#### Previous diagnoses

Participants were asked to indicate which if any conditions they had ever been formally diagnosed with on a tick list. Here, 17.2% of the participants selected learning disabilities (reduced intellectual ability and difficulty with everyday activities), 22.4% selected learning difficulties (e.g., dyslexia), 8.6% selected ADHD, 3.4% selected autism, 12.1% selected personality disorder, and 39% reported being diagnosed with a mental illness.

Additionally, 5.2% of participants stated that they had a need for social care support (defined as, e.g., help with washing or dressing); 53.4% indicated that they had physical or mental health conditions or illnesses lasting, or expecting to last, for at least 12 months; and 20.7% stated that they had a formal diagnosis for another condition not previously mentioned. Free text responses here included epilepsy, arthritis, PTSD, brain damage, IBS, high cholesterol, asthma, and gout (Table 2).

#### Assessment of needs

Overall, 52 participants completed the CANSAS-P and, 78.8% (*n* = 41) indicated that they had at least one need. In total, 71.2% of participants (*n* = 37) indicated they had at least one met need, whilst 65.4% (*n* = 34) indicated that they had at least one unmet need (see research question three for more on this). On average, the participants indicated that they had 6.8 needs overall (SD = 5.8).

#### Alcohol and drug misuse

Fifty-seven participants completed all three AUDIT-C questions. The overall mean AUDIT-C score was 3.70 (SD = 3.80). Excluding those who scored 0 (i.e., those who indicated they did not drink alcohol) the overall mean AUDIT-C score was 5.70 (SD = 3.27). Just over a third of participants completing the tool (35.1%, *n* = 20) scored 5+, indicating possible alcohol misuse. In terms of severity, for all participants, 64.9% were classed as low risk, 15.8% as increasing risk, 14% as higher risk, and 5.3% as having a possible alcohol dependence (Table 3).

Fifty-seven participants completed all items on the DAST-10. The mean score was 2.8 (SD = 3.4) and 38.6% scored three or more, indicating possible drug misuse. 47.4% of participants completing the tool indicated that they had no problems with drug misuse, 14% were identified as having a low level of severity, whilst 10.5% were classified as having a moderate level, 19.3% as having a substantial level of severity, and 8.8% as having a severe level of drug misuse (Table 3).

#### Psychological distress and mental health

56 participants completed all items on the CORE-10. The mean score was 14.3 (SD = 8.90). Overall, 17.9% scored in the healthy range, with the same proportion classified as having a low level of psychological distress. Based on the cut-off scores used by O’Connor and Morris (2019), this latter group would benefit from referral to primary care. Additionally, over two-thirds (*n* = 36) of participants scored ten or more, indicating that they had at least mild psychological distress, and would benefit from referral to secondary care (Table 4). Overall, 14.3% of participants had severe psychological distress.

Several CANSAS-P items related to mental health. As shown in Table 5, these suggested quite high levels of met and unmet needs in some areas. For example, almost a third of respondents reported ever having thoughts of self-harm as either a met (16.1%, *n* = 9) or unmet (12.5%, *n* = 7) need, and around a fifth reported hearing voices or having problems with their thoughts as a met (8.9%, *n* = 5) or unmet (12.5%, *n* = 7) need.

#### Wider determinants of health

Responses to the CANSAS-P showed that having appropriate housing was reported as a met need by 19.6% of the sample, and the same proportion reported it as an unmet need (Table 6). Likewise, support with budgeting money and receiving all the money they were entitled to were needs for over a third of participants, which were more commonly reported as met rather than unmet needs. Around a quarter of the participants indicated that getting enough food (16.1% met and 10.7% unmet) and difficulty with English (14.5% met and 10.9% unmet) were needs. Similarly, around a quarter of participants indicated having a phone and access to the internet and using the bus, tram or train as needs, but again these were more likely to be reported as met rather than unmet needs.

#### Comorbidity and complexity of needs

Table 7 shows that just over a quarter of participants (*n* = 15) scored at or above the cut-off score of 6+ on the CORE-10 (indicating at least low-level psychological distress) and 5+ on the AUDIT-C (indicating possible alcohol misuse), suggesting they have co-occurring needs in these areas. Almost a third of participants (*n* = 19) had co-occurring needs for both psychological distress and possible drug misuse (scoring 6+ on the CORE-10 and 3+ on the DAST-10). Moreover, of the 52 participants who completed all of the screening tools, 15.4% of participants (*n* = 8) scored at or above the cut-off score on all three of the screening tools (regardless of which cut-off score was used for the CORE-10). Of those eight participants, there was an average total needs score on the CANSAS-P of 12.5 (SD = 5.04) – much higher than the average total needs of the overall sample (6.82, SD = 5.81).

### What do people on probation’s patterns of service access look like?

Looking at service access, 92.7% of participants (*n* = 51) were registered with a GP. However, 48% felt like they did not see their doctor as much as they should and 50.9% did not have a dentist. Only 16.4% of participants stated that they had a psychiatrist or mental health worker, although 38% felt that they should have. Only 3.8% of participants were currently accessing social care.

Participants were also asked whether they had ever been to A&E as a patient. Attendance was high with one participant reporting attending in the last week, 4 (7.8%) in the last month, and 10 (19.6%) in the last year. A further 23 (45.1%) reported attending over a year ago, and 13 (25.5%) had not needed to attend (Table 8). None of the participants stated that they had not been but should have done.

People on probation may also access support through community sentence treatment requirements. In our sample, seven participants did not respond to this question. Of the remainder, two (3.8%) reported having a drug rehabilitation requirement; one (1.9%) reported having a mental health treatment requirement; 1 (1.9%) reported having an alcohol treatment requirement on their order and 48 (92.3%) stated they did not have any of these requirements.

### To what extent are the health and social care needs of people on probation met by existing service provision?

Further analysis of the CANSAS-P responses showed that the average number of met needs reported was 4.27 (SD = 4.26), and the average number of unmet needs reported was 2.56 (SD = 3.30). The most common unmet needs came from the items ‘Have you recently felt very sad or low?’ and ‘Are you happy with your social life?’ which applied to 23.6% and 20.0% of the participants respectively. The most frequently reported met needs were ‘Do you feel like your days are fulfilling/enjoyable?’ (39.3%), and ‘Are you happy with your social life?’ (32.7%) (Table 9).

### Does the prevalence of needs and/or the extent to which they are met vary by age, ethnicity or rurality?

#### Group differences in need

We created a series of regression models to explore whether the prevalence of needs varied by participants’ characteristics (age or ethnicity) or rurality (location of the probation office that participants reported to). In each instance, the three predictor variables were binary. Age was grouped as below 50 years versus 50 years and above; ethnicity was grouped as white versus ethnic minority; and rurality was grouped as urban versus rural. These binary variables were entered into three separate logistic regression models to predict whether the participant scored at or above the cut-off scores for the AUDIT-C (5+), the DAST-10 (3+), and the CORE-10 (6+). Additionally, linear regression models with the same three independent variables were used to predict the participants’ total number of needs as indicated by the CANSAS-P (i.e., met needs and unmet needs combined), as well as their number of met needs and unmet needs individually. In each model, the category with the highest number of observations was used as the reference baseline category (below 50; white; and urban). No significant predictors for needs were found for alcohol, drug use or psychological distress. Nor was a multiple linear regression successful in predicting overall needs, met needs or unmet needs.

#### Co-occurring needs

To determine whether age, ethnicity or rurality had any effect on co-occurring needs, we performed another binary logistic regression. A binary variable was created which indicated whether each participant scored above the cut-off score on the CORE-10, as well as scoring above the cut-off score for either the AUDIT-C or the DAST-10. As such, those with a positive score for this variable could be said to be currently experiencing psychological distress, alongside alcohol and/or drug misuse issues.

The overall model was significantly able to predict co-occurring needs (χ^2^ = 10.60, df = 3, *p* = .01). In terms of the individual variables, neither rurality (OR = 0.42, *p* = .17) nor age group (OR = 0.21, *p* = .08) were significant predictors. However, ethnicity was a significant predictor of co-occurring needs (OR = 0.14, *p* = .03, 95% CI = 0.03–0.81). This suggests that having the co-occurring needs of psychological distress as well as alcohol and/or drug misuse issues is around 86% less likely among ethnic minority participants compared to white participants. We explored this further in with a t-test, which showed that white participants were significantly more likely to have co-occurring issues (M = 54.5%, SD = 0.50) than ethnic minority participants (M = 16.7%, SD = 0.39), *t*(54) = 2.41, *p* = .02.

### What proportion of people on probation completed the survey independently and what proportion completed it with the assistance of staff within probation?

#### Sensitivity analyses

Participants completed the survey either alone (*n* = 28) or with probation staff (*n* = 26; 12 because of reading difficulties and 14 due to the pandemic) (five participants did not answer this question). To determine whether this had any effect on their responses, we performed four *t*-tests comparing the average scores on the AUDIT-C, DAST-10, CORE-10 and for a total number of needs on the CANSAS-P between those who completed it alone and those who completed it with assistance. There were no statistically significant differences, suggesting that whether a member of probation staff assisted with the completion of the survey or does not significantly influence responses.

### How acceptable is this approach to identifying the needs of probation staff and what might facilitate it becoming a routine part of probation practice?

Two members of engaging people on probation teams that had administered the survey participated in interviews.

Analysis of the interview data offered some insights into the potential challenges of this approach to identifying needs, and useful suggestions for how such an approach might best be embedded within routine practice in the future. However, the extent to which these individuals’ experiences are generalisable is unknown at this point, and future research would be needed to test their suggestions around future implementation approaches.

## Discussion

Routinely collecting data on the health and social care needs of people on probation using validated tools has numerous potential benefits. It would enable probation practitioners to identify where people may benefit from referral to health and social care partners, which would potentially support rehabilitation and reduce re-offending. If data on needs and outcomes were shared with partner agencies, then this could also inform commissioning decisions, helping to ensure investment in services and interventions that will improve health outcomes for the probation population. At present, we know that such investment is negligible (Brooker and Ramsbotham, 2014; Sirdifield et al., 2019a). Finally, it would be in line with Government policy (HM Government and NHS England, 2023) which seeks to develop the use of health outcome measures and data-sharing between agencies.

We piloted a research-informed approach to identifying the health and social care needs of people on probation, using a survey consisting of validated screening tools and additional key questions. Through this we aimed to improve understanding of the needs of the probation population and of the feasibility of employing this type of approach in routine probation practice.

We anticipated achieving a minimum response rate of 10% but fell far short of this. The difficulties with recruitment mean that we have only been able to learn a limited amount about the needs of the probation population from this study.

However, there are key headlines to our findings which demonstrate the potential of this approach if it were used more widely. The sample characteristics were similar to those of the overall probation population although our sample might be older than the population. Over half of the sample reported having physical or mental health conditions or illnesses lasting, or expecting to last, for at least 12 months. This compares to 40% of adult men reporting a longstanding illness in the Health Survey for England (2019) (NHS Digital, 2020). In addition, 39.6% had been either diagnosed with a learning difficulty or disability. Just over a fifth of our sample reported a formal diagnosis for a condition not listed on the survey, including epilepsy, irritable bowel syndrome and arthritis. Over a third of the sample had either an alcohol or drug use issue. The CORE-10 was used to measure psychological distress. A small proportion (17.9%) scored above the threshold for minor psychological distress a cut-off for referral to primary care; whilst over two-thirds scored at the higher level (10+), meaning that a referral to specialist secondary mental health services was indicated.

Nearly 60% of participants had at least one unmet need, with 23.6% reporting that recently feeling sad or low was an unmet need. There was a high complexity of issues within the sample population – using the higher cut-off score of 10+ on the CORE-10, 23 participants (44% of those completing all three tools) would benefit from referral to secondary mental health services and had a possible alcohol and/or drug misuse problem, i.e., a dual diagnosis. Currently, many services are designed to cater for just one problem (Richards, 2020), but these figures show how conducting assessments during probation could evidence the need to commission services that cater for complex needs.

The data on service use showed an encouragingly high level of GP registration (92.7%) but only 49.1% were registered with a dentist. Over a quarter had visited Accident and Emergency in the last year. Many aspects of these data will have changed, post-covid, for the general population so speculation about the possible inferences to be drawn for people on probation is not straightforward. Also, this study is based on a small, non-random sample, and consequently, future research would be needed to establish the generalisability of the findings to the national probation caseload.

A helpful finding is that at least in our sample, there was no significant variation in reporting of needs between those who completed the survey alone, and those who completed it with a member of probation staff. This has implications for future work of this nature.

The study has provided some learning about the acceptability of the approach that we piloted, and how best to enable the recording of data about health and social care needs as a routine part of probation practice, but further research is needed to understand this, and the potential impact of adopting such an approach in more detail. In Table 10, we detail every aspect of the study and explain the challenges that arose. The table shows that despite our best intentions recruiting cases and interviewees from the probation service proved difficult. Recruitment appears to have been impacted by the fact that the project was conducted at a time when the probation service was continuing to experience pressures following the COVID-19 pandemic, was recovering after the process of being unified following unsuccessful privatisation, and had lost large numbers of staff with the remainder experiencing low morale and high caseloads (Tidmarsh, 2020).

All of the measures were short and appropriate for use in probation (see, e.g., Brooker et al., 2022). Apart from the CANSAS-P, they were all completed by over 90% of the sample (Table 11).

Based on feedback from staff and learning throughout, we recommend a reduced version of the survey.^5^ Demographic data should already be routinely recorded, and those answering ‘no’ to the first questions on the AUDIT-C or DAST-10 could skip the remainder of the tool with alcohol and/or drug misuse consequently being recorded as ‘not an issue’. Three questions from the CANSAS-P have been included as starting points for a discussion around any potential need to see a GP or access social care. However, the approach to recording responses has been simplified. Ideally, these assessment tools would be integrated into case management systems for completion over several appointments. The tools would be accompanied by processes and procedures for signposting and referral to appropriate services where people consent to this and would benefit from it, together with recording of outcomes data.

If completed routinely, this would vastly improve the evidence base on the needs of the probation population and generate data that could be shared at the aggregate level with commissioners. Such data could also be interrogated to explore differences between groups and thereby develop a more nuanced understanding of the needs that services should address. Evidencing the needs of the probation population in this way would be the first step towards improving service provision to address the needs of this population. As stated earlier, further research is needed to investigate the acceptability of such an approach in probation practice. With support from probation, we are currently exploring further testing and evaluation of this in future research.

As indicated at the start of this article, further action also needs to flow from this, for example, to ensure that sufficient resources are available to commission the treatment and support that is needed, establish clear pathways into care from probation, update probation’s health and social care strategy and to increase our understanding of the quality of care received and the efficacy of different interventions for this group. Again, we intend to explore mechanisms to support this in future research.

## Conclusion

We tested a research-informed approach to identifying and recording health and social care needs in probation. Recruitment to the study was problematic and consequently further research would be needed to establish the generalisability of our findings on the prevalence of health and social care needs. Based on feedback from participants in the study and our experience of conducting it, we recommend a reduced version of our survey which could be incorporated into probation systems for further testing and routine use in practice. Whilst the acceptability and impact of this approach require further study, we believe it would increase understanding of the current health and social care needs of and longer-term trends in the needs of people on probation and create data that could be used to inform commissioning decisions. This would be a first step towards achieving appropriate and accessible service provision for this group.

## Figures and Tables

**Table 1:** Participant characteristics for the survey sample and overall probation caseload.

		Survey sample (*n* = 59)			Overall probation caseload* (*n* = 152,870)	
		*N*	*%*		*N*	*%*
Age						
	Less than 50 years	47	79.7		134,945	88.3
	50 years and above	10	17.0		17,925	11.7
	Missing/prefer not to say	2	3.4		0	0.0
Ethnicity						
	White	47	79.7		114,259	75.0
	Ethnic minority	12	20.3		32,818	21.0
	Missing/prefer not to say	0	0.0		5793	4.0

* Figures for male offenders supervised in the community by the probation service in England and Wales on 30 September 2022, provided by the Ministry of Justice.

**Table 2:** Previous diagnoses.

Previous diagnosis	*N*	%
Learning disability	10	17.2
Learning difficulty	13	22.4
ADHD	5	8.6
Autism	2	3.4
Social care support	3	5.2
Personality disorder	7	12.1
Mental illness	23	39.0
Any other health conditions	12	20.7
Any physical or mental health conditions or illnesses lasting or expecting to last at least 12 months	31	53.4

**Table 3:** Severity of alcohol and drug misuse.

AUDIT-C and DAST-10 classifications		*N*	%
AUDIT-C			
	Low risk (0–4)	37	64.9
	Increasing risk (5–7)	9	15.8
	Higher risk (8–10)	8	14.0
	Possible alcohol dependence (11–12)	3	5.3
	Scoring above cut-off score (5+)	20	35.1
DAST-10			
	No problems (0)	27	47.4
	Low level (1–2)	8	14.0
	Moderate (3–5)	6	10.5
	Substantial (6–8)	11	19.3
	Severe (9–10)	5	8.8
	Scoring above cut-off score (3+)	22	38.6

AUDIT-C: Alcohol Use Disorders Identification Test; DAST-10: Drug Abuse Screening Test.

**Table 4:** Severity of psychological distress (CORE-10).

CORE-10 classifications	*N*	%
Healthy (0–5)	10	17.9
Low level (6–9)	10	17.9
Mild psychological distress (10–14)	9	16.1
Moderate distress (15–19)	12	21.4
Moderately severe distress (20–24)	7	12.5
Severe psychological distress (25+)	8	14.3
Scoring at or above cut-off score of 6	46	82.1
Scoring at or above cut-off score of 10	36	64.3

CORE-10: Clinical Outcomes in Routine Evaluation.

**Table 5:** CANSAS-P responses related to mental health.

CANSAS-P item	No need (not a problem for me)	Met need	Unmet need	Don’t want to answer
Do you ever hear voices or have problems with your thoughts?	43 (76.8%)	5 (8.9%)	7 (12.5%)	1 (1.8%)
Have you recently felt very sad or low?	24 (43.6%)	17 (30.9%)	13 (23.6%)	1 (1.8%)
Do you ever have thoughts of harming yourself?	38 (67.9%)	9 (16.1%)	7 (12.5%)	2 (3.6%)
Do you think you could be a danger to other people’s safety?	45 (81.8%)	5 (9.1%)	2 (3.6%)	3 (5.5%)

CANSAS-P: Camberwell Assessment of Need Short Appraisal Schedule.

**Table 6:** CANSAS-P item responses related to wider determinants of health.

CANSAS-P item	No need (not a problem for me)	Met need	Unmet need	Don’t want to answer
Do you have appropriate housing?	34 (60.7%)	11 (19.6%)	11 (19.6%)	0 (0.0%)
Do you get enough food?	41 (73.2%)	9 (16.1%)	6 (10.7%)	0 (0.0%)
Are you able to look after your home?	43 (78.2%)	6 (10.9%)	6 (10.9%)	0 (0.0%)
Do you have problems keeping clean and tidy?	44 (78.6%)	9 (16.1%)	3 (5.4%)	0 (0.0%)
Do you have any difficulty in reading, writing, or understanding English?	40 (72.7%)	8 (14.5%)	6 (10.9%)	1 (1.8%)
Do you have a phone and access to the internet?	39 (70.9%)	13 (23.6%)	2 (3.6%)	1 (1.8%)
How do you find the bus, tram, or train?	38 (71.7%)	11 (20.8%)	4 (7.5%)	0 (0.0%)
Do you need support with budgeting your money?	33 (60.0%)	13 (23.6%)	8 (14.5%)	1 (1.8%)
Are you getting all the money you are entitled to?	32 (58.2%)	15 (27.3%)	8 (14.5%)	0 (0.0%)

CANSAS-P: Camberwell Assessment of Need Short Appraisal Schedule.

**Table 7:** Co-occurring needs.

Scoring at or above the cut-off score		AUDIT-C (5+)			DAST-10 (3+)
		Yes	No		Yes	No
CORE-10 (6+)	Yes	15 (27.8%)	29 (53.7%)		19 (35.2%)	25 (46.3%)
	No	4 (7.4%)	6 (11.1%)		2 (3.7%)	8 (14.8%)
CORE-10 (10+)	Yes	15 (27.8%)	20 (37.0%)		16 (29.6%)	18 (33.3%)
	No	4 (7.4%)	15 (27.8%)		5 (9.3%)	15 (27.8%)

AUDIT-C: Alcohol Use Disorders Identification Test; DAST-10: Drug Abuse Screening Test; CORE-10: Clinical Outcomes in Routine Evaluation.

**Table 8:** Service access.

Service access responses	*N*	%
Registered with a GP	51	92.7
Sees their GP enough	26	52.0
Has a dentist	27	49.1
Not registered with a dentist and reports difficulty registering	12	50.0*
Has a psychiatrist or mental health worker	9	16.4
Thinks they should have a psychiatrist or mental health worker	19	38.0
Been to A&E as a patient:		
Yes in the last week	1	2.0
Yes in the last month	4	7.8
Yes in the last year	10	19.6
Yes over a year ago	23	45.1
No I haven’t needed to	13	25.5
Currently accessing social care	2	3.8

* Percentage of those not registered with a dentist.

**Table 9:** Most frequently indicated met and unmet needs.

		*N*	%
**Met needs**			
	Do you feel like your days are fulfilling/enjoyable?	22	39.3
	Are you happy with your social life?	18	32.7
	Have you recently felt very sad or low?	17	30.9
	Are you getting all the money you are entitled to?	15	27.3
	How well do you feel physically?	14	25.0
**Unmet Needs**			
	Have you recently felt very sad or low?	13	23.6
	Are you happy with your social life?	11	20.0
	Do you have appropriate housing?	11	19.6
	Are you in a fulfilling relationship?	10	18.2
	How well do you feel physically?	9	16.1

**Table 10:** Original objectives and associated progress, challenges and considerations for future work.

The original research objective	Progress and challenges
Pilot the survey via Revolving Doors Agency with 5–10 people with lived experience and make adjustments if needed based on feedback.	Completed successfully.
Administer the survey via probation staff over four PDUs, and achieve a minimum response rate of 10%.	Survey rolled out to four PDUs, but we didn’t achieve this response rate. This was largely due to resourcing issues and competing pressures within probation. Interview data suggest that we should slightly reduce the survey length, build the tools into probation systems with prompts for staff to complete them, and ask staff to complete them over several appointments rather than all in one go.
Conduct a sensitivity analysis on the impact of whether assistance was required to complete the survey.	Completed successfully.
Use demographic data to determine the extent to which survey respondents reflect the population within the national probation caseload.	Completed successfully.
Weight the data to better represent the national population in terms of its demographic profile using propensity score weighting.	Data weighted but no significant differences were detected. Weighted analysis is available as a technical appendix on request.
Analyse data using descriptive statistics to show (a) the prevalence of needs and (b) patterns of service access and the extent to which needs are being met.	Completed successfully.
Explore variation by PDU characteristics (urban/rural) and investigate what effect individual demographics have on health and wellbeing outcomes – comparing sex, age, ethnicity, and past diagnosis to the health and wellbeing outcomes measured on the AUDIT-C, DAST-10, CORE-10 and CANSAS-P.	Completed successfully, except only for male participants.
Interview 5–10 staff about their experience of administering the survey – use this to inform a proposed implementation model that will be created at the end of the project	Two staff were interviewed.

AUDIT-C: Alcohol Use Disorders Identification Test; DAST-10: Drug Abuse Screening Test; CORE-10: Clinical Outcomes in Routine Evaluation; PDU: Probation Delivery Unit; CANSAS-P: Camberwell Assessment of Need Short Appraisal Schedule.

**Table 11:** Potential future approach to identifying health and social care needs.

Measure	Proportion of the sample that completed this measure	Future utility
Do you have any physical or mental health conditions or illnesses lasting or expecting to last 12 months or more?	98.3% (*n* = 58)	High
AUDIT-C	96.6% (*n* = 57)	High
DAST-10	96.6% (*n* = 57)	High
CORE-10	94.9% (*n* = 56)	High
CANSAS-P	88.1% (*n* = 52)	Low
Do you have a doctor (GP)?	93.2% (*n* = 55)	High
Do you have a dentist?	93.2% (*n* = 55)	High
Do you have a psychiatrist or mental health worker?	93.2% (*n* = 55)	High

AUDIT-C: Alcohol Use Disorders Identification Test; DAST-10: Drug Abuse Screening Test; CORE-10: Clinical Outcomes in Routine Evaluation; CANSAS-P: Camberwell Assessment of Need Short Appraisal Schedule.

## Notes

1 The NPS was dissolved in 2021 when the probation service in England and Wales (which had been split into the NPS and Community Rehabilitation Companies) was unified.

2 See https://cks.nice.org.uk/topics/alcohol-problem-drinking/diagnosis/how-to-screen/

3
https://www.corc.uk.net/media/2311/perinatal-roms-manual-a4-final-print-december-2019.pdf

4
https://www.coresystemtrust.org.uk/home/instruments/core-10-information/

5 This is available from the corresponding author on request.
